# High-resolution phenotypic profiling of natural products-induced effects on the single-cell level

**DOI:** 10.1038/srep44472

**Published:** 2017-03-15

**Authors:** Stephan Kremb, Christian R. Voolstra

**Affiliations:** 1 Red Sea Research Center, Division of Biological and Environmental Science and Engineering (BESE), King Abdullah University of Science and Technology (KAUST), Thuwal 23955-6900, Saudi Arabia

## Abstract

Natural products (NPs) are highly evolved molecules making them a valuable resource for new therapeutics. Here we demonstrate the usefulness of broad-spectrum phenotypic profiling of NP-induced perturbations on single cells with imaging-based High-Content Screening to inform on physiology, mechanisms-of-actions, and multi-level toxicity. Our technology platform aims at broad applicability using a comprehensive marker panel with standardized settings streamlined towards an easy implementation in laboratories dedicated to natural products research.

With improved strategies of natural products discovery and rapidly evolving technologies of analytical chemistry, there is a steadily growing number of novel and unique chemical scaffolds that hold promise as lead compounds for biomedical applications^1^,^2^,^3^. In contrast, there is a remarkable gap of knowledge on biological activities on a large proportion of natural products (NPs)^4^. Traditionally, most studies on biological activities of NPs are focused on single molecular targets or general toxicity as a marker of potential anti-neoplastic activity^5^,^6^,^7^. However, it is becoming increasingly clear that more holistic approaches are needed to fully explore the potential of NPs and to provide strategies for the prioritization of lead compounds^8^. To this end, imaging-based High-Content Screening (HCS) has emerged as a promising tool for primary screening of small molecules, which provides a powerful strategy for untargeted biological profiling of chemical compounds and to predict compound-related mechanisms of activity^9^,^10^,^11^,^12^,^13^,^14^,^15^,^16^. However, most of the current studies that employ HCS for the characterization of NPs focus on a limited set of cellular markers. In this study, we developed a more versatile approach for the comprehensive characterization and classification of NPs according to four major categories of relevant biological information: (1) compound-induced perturbations of multiple cellular processes, (2) multi-level toxicity, (3) structure-activity relationships (SAR), and (4) mechanism of action (MOA) and potential molecular targets or off-target effects.

## Results and Discussion

**Figure 1:**
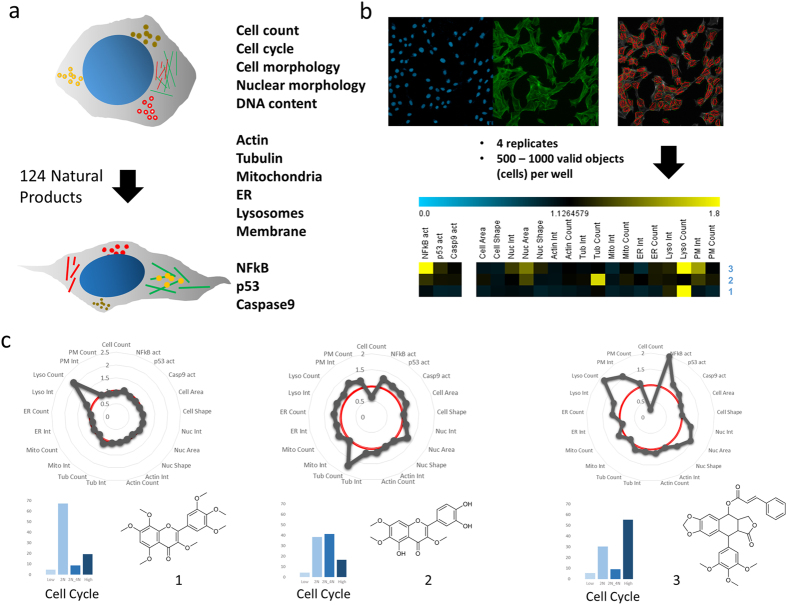
Broad-spectrum High-Content Screening technology platform providing high-resolution cytological profiling. (**a**) A combination of 14 cellular markers informs on compound-induced perturbations, including all major organelles as well as components of important regulatory pathways. (**b**) The generation of cytological profiles involves fluorescent staining of cellular targets, imaging, and subsequent quantitative analysis of selected features from 500–1000 valid objects (i.e., cells). An overall number of 134 cellular features is extracted to generate high-resolution cytological profiles. These are reduced to 20 ‘core’ features for straight-forward analysis and convenient visualization. (**c**) Cytological profiles composed of 20 core features of three natural products are shown as polar plots, including two flavones (1 = 3,3′,4′,5,5′,7,8-Heptamethoxyflavone; 2 = 3′,4′,5,6,7-Pentahydroxy-3-methoxyflavone) and a derivative of podophyllotoxin (3). Bar plots indicate percentages of cells in each phase of the cell cycle: Low = damaged or apoptotic cells (low DNA content); 2N = cells in G0/G1 phase; 2N_4N = cells in S phase; High = cells in M phase and higher ploidy.

Our technology platform comprises a combination of 14 cellular markers that inform on key components of cell physiology, including all major organelles as well as important regulatory pathways to generate high-resolution cytological profiles (CPs) that serve as unique fingerprints for compound-induced perturbations (Fig. 1a). The process of cytological profiling involves fluorescent staining of specific cellular targets, imaging, and subsequent quantitative analysis of selected features from 500–1000 valid objects (i.e., cells) (Fig. 1b). Cellular descriptors or features (derived from means of valid objects and related to a control treatment) depict either morphological characteristics or fluorescence intensities of the selected cellular target and are resolved according to their intracellular location. In order to facilitate visualization, we reduced 134 cellular features that describe CPs to a set of 20 ‘core’ features focusing on descriptive and easy-to-understand parameters (Supplementary Table 1). As a proof-of-concept, we tested a collection of 124 poorly characterized and chemically diverse NPs (Supplementary Table 2), including three series of closely related molecules, for the assessment of structure-activity relationships (Supplementary Fig. 1). Cytological profiles retrieved from the NP collection were compared against a library of 720 single bioactive reference compounds selected from the LOPAC^®^1280 library of pharmacologically active compounds (Sigma Aldrich, international version, http://www.sigmaaldrich.com/life-science/cell-biology/bioactive-small-molecules/lopac1280-navigator.html) with established MOAs to predict putative biological targets based on the assertion that similar phenotypic responses are inferred to have similar MOAs and putatively similar underlying structural principles^11^,^12^,^13^. We found a high degree of distinctive variation for all of the core features in the reference compound library (Supplementary Fig. 2a) and for the poorly characterized NP collection (Supplementary Fig. 2b) demonstrating the ability of these descriptors to capture the complexity of cytological perturbations upon NP treatment. Almost all of the tested compounds affected at least one cellular marker, highlighting the significance of using a broad spectrum of markers to describe compound-related perturbations (Supplementary Fig. 3).

A closer look at a set of three compounds from the collection of poorly characterized NPs, i.e. a pair of two structurally related flavones and a derivative of podophyllotoxin, further illustrates some of the major benefits of broad spectrum cytological profiling to assess compound-induced perturbations (Fig. 1c). The first Flavone (1) shows no reduction in cell numbers and only a single strong effect on the number of lysosomes, while all other markers remain unaffected. In contrast, the second flavone (2) shows strongly reduced cell counts and moderate perturbations of multiple cellular processes leading to an S phase arrest. The CP resulting from a derivative of podophyllotoxin (3) with severe cell loss and a strong mitotic arrest reveals multiple perturbations, including NFkB activation, nuclear effects as well as severe effects on lysosomal and plasma membrane markers. These findings highlight: (i) simultaneous analysis of a multitude of cellular markers in one experimental setup can greatly assist to understand the interplay of processes and to assess mechanisms of toxicity, (ii) compounds that show perturbations only on few or single markers while at the same time leaving other processes unaffected may provide good starting points in the search for more selective drug candidates, and (iii) the finding that small structural variations of a molecule can have pronounced effects on multiple cellular processes opens up the possibility of comprehensive and more physiologically relevant cell-based SAR studies.

Compound toxicity is a major factor that determines the suitability of a molecule for further evaluation in bioactivity studies. Most studies on bioactivity of NPs used unidimensional measures of cell viability (e.g., MTT test)^17^ or cell density determination (e.g., Sulforhodamine B colorimetric assay)^7^. However, compound toxicity is often a combination of multiple mechanisms that ultimately compromise cell health or lead to cell death. Thus, cytological profiling provides a strong foundation for the assessment of the toxic potential of NPs. For our technology platform, we evaluated cell loss, a widely used marker, to assess cytotoxicity. Indeed, a considerable number of NPs caused a pronounced reduction of cell counts demonstrating the suitability of cell loss to assess toxic potential (Supplementary Fig. 4a). For instance, a closer look at candidate toxic compounds revealed a group of 16 closely related derivatives of podophyllotoxin with highly similar cytological profiles, but strong variations of cytoskeletal markers (Supplementary Fig. 4b). Importantly, reduced cell counts correlated largely with NFkB activation. A second group of compounds that caused significant cell loss included molecules from various different structural classes (Supplementary Fig. 4c). Again, the extent of cell loss was correlated with activation of NFkB, but the underlying mechanisms that lead to NFkB activation presumably differ between compounds. Interestingly, a derivative of adenosine (6-phenyladenosine) demonstrated strong NFkB activation with 40% cell loss, but almost no effect on any other marker.

**Figure 2:**
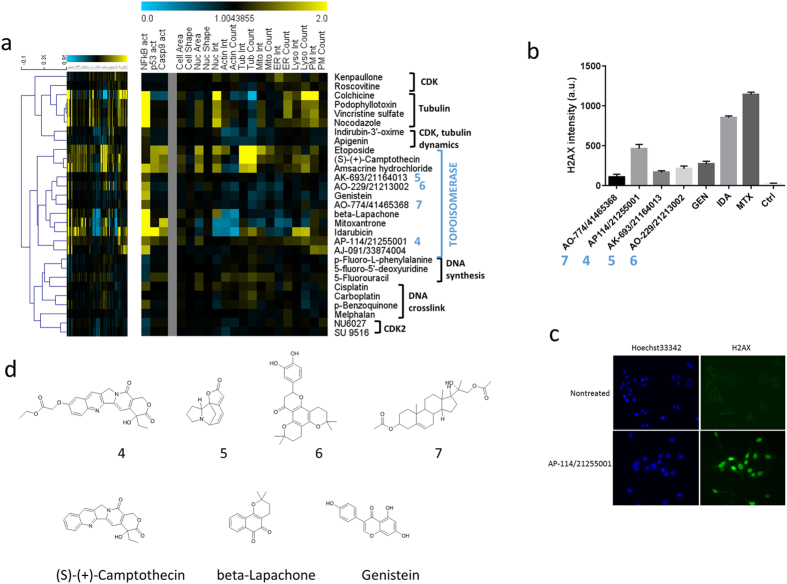
Prediction and validation of biological targets/mechanism of action (MOA) of antineoplastic compounds. (**a**) Hierarchical clustering of cytolological profiles composed of 20 core features of a group of 26 anti-neoplastic compounds reveals various distinct functional sub-groups that clearly reflect different molecular targets and MOAs. Colors indicate positive (yellow) or negative (blue) deviation from the mean of untreated control cells (value = 1). Full cytological profiles were used for clustering (left). Cytological profiles showing a reduced set of 20 core markers are displayed for easier visualization of affected processes and a vertical grey bar was used to separate between regulatory (i.e. NFkB, p53, and caspase 9 activation) and other cellular markers. Colors indicate positive (yellow) or negative (blue) deviation from the mean of untreated control cells (value = 1). The dendrogram depicts distances between individual cytological profiles based on Spearman rank correlation. (**b**) Four candidate natural products and three reference compounds were tested for phosphorylation of H2AX in a cell based assay to assess DNA double strand breaks caused by topoisomerase II poisons (control shows no effects). (**c**) Detection of phospho-H2AX in HeLa cells treated with vehicle control (non-treated) or with 20 μM of compound 4 (see d) for 6 hours. (**d**) Structures of selected natural products and reference compounds of the topoisomerase cluster from (**a**): derivative of 10-hydroxycamptothecin (4), Norsecurinine (5), Dorsmanin E (6), 2-(3-Acetoxy-10,13-dimethyl-2,3,4,7,8,9,10,11,12,13,14,15,16,17-tetradecahydro-1H-cyclopenta[a]-phenanthren-17-yl)-2-hydroxypropyl acetate (7), (S)-(+)-Camptothecin, beta-Lapachone, and Genistein.

Recent studies clearly demonstrate that phenotypic profiling is a powerful tool to predict mechanisms of action (MOA) of candidate molecules based on the assumption that compounds with similar phenotypic responses have similar biological targets or MOAs^11^,^12^,^13^. Similarity between CPs of reference compounds with assigned MOAs and candidate compounds from our collection of poorly characterized NPs were assessed via hierarchical clustering^18^, which resulted in several groups of closely matching reference compound/NP pairs. In the following, we highlight three examples from different target classes by focusing on the underlying cytological as well as structural principles and present follow-up results that substantiate the predictions of potential biological targets. In all cases, reference compounds could be reliably resolved into functional groups supporting the significance of MOA prediction by our technology platform. A cluster of 26 anti-neoplastic compounds revealed various distinct functional sub-groups that clearly reflect different molecular targets/MOAs (Fig. 2a). These include compounds acting on the DNA level (e.g., DNA synthesis, DNA binding, DNA crosslinking) as well as compounds that target cyclin-dependent kinases (CDKs), tubulin polymerization, and topoisomerase inhibitory activity. Five compounds were found to match with a set of topoisomerase poisons/inhibitors, of which we investigated four further. CPs of compounds 5, 6, and 7 (Fig. 2d) exhibited high similarities to genistein, an isoflavone with topoisomerase II inhibitory activity^19^, whereas a derivative of 10-hydroxycamptothecin (compound 4, Fig. 2d) shared similarity in regard to CP and chemical structure with the reference compound (S)-(+)-Camptothecin, a quinolone alkaloid that inhibits topoisomerase I^20^. Topoisomerase poisons/inhibitors are well known to induce DNA double strand breaks, which were found to subsequently lead to rapid phosphorylation of H2AX at Ser139 by PI3K-like kinases^21^,^22^. In fact, all four candidate compounds significantly induced phosphorylated H2AX after 6 hours of treatment to an extent comparable to genistein (Fig. 2b,c). We identified another NP candidate molecule, a derivative of the plant-derived kenunsanone B, matching closely with two protease inhibitors, E-64 and nelfinavir (NLV) that is further discussed in the supplemental section (Supplementary Text 1, Supplementary Fig. 5). Further, a set of structurally diverse NP candidate molecules were found to cluster with a large group of neurotransmitter (NT)-related reference compounds, including NT receptor antagonists, NT re-/uptake inhibitors, or ion channel interfering compounds (Supplementary Text 2, Supplementary Fig. 6a). Notably, most of the candidate molecules show clear structural similarities to several of the reference compounds (Supplementary Fig. 6b). Several drugs found in this cluster are known to accumulate in lysosomes and to increase lysosomal counts (e.g. fluoxetine, chlorpromazine, chloroquine, clomipramine or desipramine) due to basic moieties found in these lipophilic or amphiphilic compounds. Once inside the acidic environment of the lysosome, these molecules become protonated and trapped in the organelle^23^. Most of the candidate compounds found in this cluster also contain a basic nitrogen and therefore probably mimic the strong lysosomal signature of the NT-related compounds.

**Figure 3:**
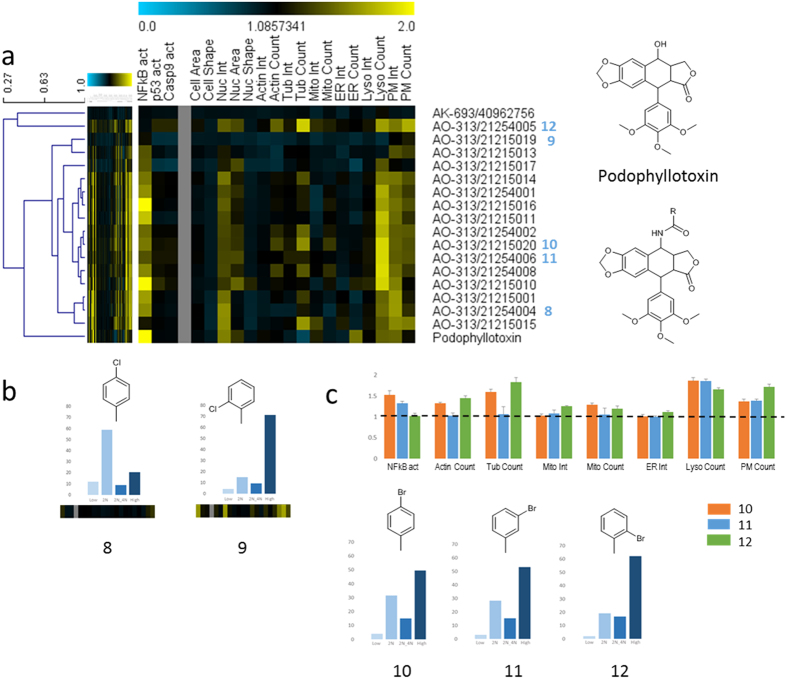
High-resolution cell-based structure-activity profiling. (**a**) Hierarchical clustering of cytological profiles of a group of 16 closely related derivatives of podophyllotoxin reveals highly similar patterns of activity on a set of markers as well as pronounced individual differences. Full cytological profiles were used for clustering (left). Cytological profiles showing a reduced set of 20 core markers are displayed for easier visualization of affected processes and a vertical grey bar was used to separate between regulatory (i.e. NFkB, p53, and caspase 9 activation) and other cellular markers. Colors indicate positive (yellow) or negative (blue) deviation from the mean of untreated control cells (value = 1). The dendrogram depicts distances between individual cytological profiles based on Pearson correlation. (**b**) Comparison of cell cycle profiles (bar plots) and cytological profiles (heat map) of two chlorinated derivatives distinguished only by the position of chlorine in the phenyl ring. Cell cycle profiles indicate percentages of cells in each phase of the cell cycle: Low = damaged or apoptotic cells (low DNA content); 2N = cells in G0/G1 phase; 2N_4N = cells in S phase; High = cells M phase and higher ploidy. (**c**) Comparison of cytological profiles (upper bar plots) and cell cycle profiles (lower bar plots) of three brominated derivatives distinguished only by the position of bromine in the phenyl ring. Most pronounced differences of the three compounds (i.e., 10, 11, 12) in the cytological profiles are represented in the colored bar plot. Dashed line indicates control treatment levels (i.e., 1). Cell cycle profiles indicate percentages of cells in each phase of the cell cycle: Low = damaged or apoptotic cells (low DNA content); 2N = cells in G0/G1 phase; 2N_4N = cells in S phase; High = cells in M phase and higher ploidy. Chemical structures show podophyllotoxin and a scaffold of the derivatives of podophyllotoxin that were used in this study.

Related chemical structures have been found to result in largely similar cellular phenotypes in various studies^11^,^12^,^13^. This opens up the possibility for Imaging-based HCS to study SARs on a cellular level, which might greatly assist in the optimization of lead molecules side-by-side with single-target assays that are typically used to optimize compound potency and specificity. Here, we studied cell-based SARs of a series of 17 closely related derivatives of podophyllotoxin (PPT), a non-alkaloid lignan, which has several clinical applications and showed potential as an antitumour agent^24^. However, its strong toxicity and adverse effects has limited the use of PPT as a treatment and led to the development of a variety of semisynthetic derivatives with improved pharmacological properties such as etoposide or teniposide^24^. On our technology platform, PPT and most of the 17 closely related derivatives showed similar effects on a set of core markers, including activation of NFkB, increased nuclear intensity, lysosomal counts, and effects on the plasma membrane (Fig. 3a). Apart from these common features, most compounds were found to exhibit perturbations of cytoskeletal markers with about half of the compounds showing strong induction of either tubulin or actin, or both. Of note, the CPs of PPT and its derivative etoposide exhibited marked differences on tubulin markers as well as NFkB, p53, and caspsase activation (Fig. 3a). This is in concordance with the different MOAs of both molecules, which is also reflected by the clustering of both molecules into different groups (Fig. 3a)^25^. A closer look at a set of halogenated derivatives differing only in the position of a chlorine or bromine in the phenyl ring reveals strong differential effects on multiple cellular markers (Fig. 3). Whereas a para-chlorinated derivative (compound 8, Fig. 3b) shows almost no effect on any of the cellular markers, the corresponding ortho-substituted derivative (compound 9, Fig. 3b) exhibits a strong M phase arrest accompanied by strong activation of NFkB and perturbation of nuclear intensity, lysosomal counts, and plasma membrane (Fig. 3b). Similarly, a series of three brominated molecules (compounds 10, 11, 12, Fig. 3c) exhibited M phase arrest with increasing extent depending on the position of the bromine. Interestingly, NFkB activation decreases in the same order with no activation for the ortho-substituted molecule (compound 12, Fig. 3c). Conversely, this derivative shows strongest perturbations for a number of other markers. These data demonstrate that related chemical structures often lead to similar phenotypic responses. Conversely, even minor structural changes may have dramatic effects on multiple cellular processes. Our high resolution cytological profiling approach is able to capture these changes in great detail highlighting the significance of using a broad panel of cellular markers. An additional example of cell-based SARs of 18 NPs with steroid scaffolds is available as supplemental material (Supplementary Text 3, Supplementary Fig.7). A recent study on triads of constitutional isomers of synthetic compounds using a similar high-content phenotypic profiling approach also found dramatic changes of biological activity caused by small structural alterations of the same scaffold^26^. This supports the finding that chemical similarity often correlates poorly with biological activity and that structurally diversity of a compound collection does not necessarily reflect diversity of biological activity. Instead, it was suggested to create “performance-diverse” compound collections (similar to our approach followed here) based on broad biological activity screens on cellular models in order to enhance the identification of novel mechanisms-of-action or biological targets^27^.

## Conclusions

In conclusion, high-resolution phenotypic profiling using a versatile set of cellular markers offers a path to comprehensively study bioactivity of NPs by providing (1) characterization of natural products-induced effects on the single-cell level, (2) prediction of biological targets, (3) mechanisms of toxicity, and (4) elucidation of structure-activity-relationships. We demonstrate that the use of reference compound profiles obtained from single molecules of known structure with assigned biological activity allows for the straightforward prediction of molecular targets from so far uncharacterized natural products. This should help to narrow the gap of knowledge on NP bioactivity and might greatly assist in prioritization of NPs for further studies. Moreover, imaging-based HCS can assist medicinal chemistry in optimizing promising lead compounds. Importantly, our technology platform aims at broad applicability through the use of the widely used HeLa cell line, commercially available reagents, standardized patterns of cell descriptors, and Sigma Aldrich’s LOPAC1280 chemical library as a source of reference compounds and is streamlined towards an easy implementation in laboratories dedicated to natural products research.

## Methods

### Chemical compounds

720 single molecule reference compounds were selected from the LOPAC^®^1280 library of pharmacologically active compounds (Sigma Aldrich, international version, http://www.sigmaaldrich.com/life-science/cell-biology/bioactive-small-molecules/lopac1280-navigator.html). Natural products were purchased from Specs (Specs.net).

### Cell culture

HeLa cells (parental HeLa cell line, ATCC^®^ CCL-2™) were kept under standard conditions at 37 °C in 5% CO_2_ in Dulbecco’s modified Eagle medium (DMEM containing GlutaMAX-1; Life Technologies) supplemented with 10% fetal bovine serum (Life Technologies) and 1% antibiotic-antimycotic solution (Life Technologies).

### High-Content Screening (HCS) Technology Platform

HeLa cells were transferred to 384-well plates at an approximate density of 2,000 cells per well in a volume of 25 μl of cell culture medium and kept under standard conditions for 24 hours. Cells were treated with 25 μl of re-dissolved fractions in 4 replicates. Only single-dose experiments were conducted at a concentration of 10 μM. 24 hours after treatment, four different cell-staining protocols (panels) were used to stain for 10 cellular targets. In all cases, cells were fixed with 4% formaldehyde for 20 minutes. For permeabilization, blocking, and washing steps, HCS-optimized reagents were used (Cellomics HCS reagents Wash Buffer (WB), Wash Buffer II (WBII), Blocking Buffer (BB) and Permeabilization Buffer (PB), Thermo Fisher Scientific). Following the last staining, all plates were washed three times with WB, sealed and stored at 4 °C until imaging. **Panel 1 (Actin/Tubulin):** Fixed cells were permeabilized for 15 minutes and blocked for 15 minutes. 12.5 μl of the primary staining solution containing 3.6 μl/ml phalloidin-FITC (Sigma Aldrich) and 1.3 μl/ml of beta-tubulin antibody (Thermo Fisher Scientific) were added per well for 1 hour. After two washing steps with BB, 12.5 μl of the secondary staining solution was added (1:500 GAM-DyLight 550, Thermo Fisher Scientific, in BB) for 1 hour. Subsequently, cells were washed 3 times with WB and nuclei were stained with 0.1 μl/ml of Hoechst33342 (Thermo Fisher Scientific). **Panel 2 (ER/Lysosomes/Membrane)**: Cells were incubated with stains for the ER (1 μl/ml, ER-Tracker Blue-White DPX, Life Technologies) and lysosomes (0.2 μl/ml, LysoTracker Red DND-99, Life Technologies) in pre-warmed cell culture medium for 30 minutes under standard conditions. After fixation, cells were washed twice with WB and incubated with a solution of a labelled wheat germ agglutinin (5 μl/ml, Wheat Germ Agglutinin, Alexa Fluor^®^ 488 Conjugate, Life Technologies) for 10 minutes. **Panel 3 (Mitochondria/NFkB):** Cells were incubated with a solution of mitochondrial dye (0.17 μl/ml, MitoTracker^®^ Orange CMTMRos, Life Technologies) in cell culture medium for 30 minutes under standard cell culture conditions. After fixation, cells were permeabilized, washed twice with WB, and incubated with the primary staining solution, including the antibody for NFkB (Thermo Fisher Scientific) for 1 hour. After removal of the primary antibody solution, cells were incubated with WBII for 15 minutes, washed twice with WB, and incubated with the secondary staining solution (1:500 GAR-DyLight 550, Thermo Fisher Scientific, in WB) for 1 hour. Subsequently, cells were incubated with WBII for 10 minutes and stained with a solution of Hoechst33342 (0.1 μl/ml, Thermo Fisher Scientific) afterwards for another 10 minutes. **Panel 4 (p53/Caspase 9)**: After fixation, cells were permeabilized for 17 minutes, washed twice with WB, and blocked for 30 minutes. After removal of BB, cells were incubated with the primary antibody solution (5.5 μl/ml of p53 antibody and 1.5 μl/ml of caspase 9 antibody, both Thermo Fisher Scientific, in blocking buffer) for 1 hour. After two washing steps with WBII and one washing step with WB, the secondary staining solution (1:500 GAR-DyLight 488 and 1:500 GAM-DyLight 550, both Thermo Fisher Scientific, in WB) was added for 1 hour. After removal of the staining solution, cells were washed once with WBII and stained with Hoechst33342 for 10 minutes.

For High-Content Analysis, the Cellomics ArrayScan VTI (Thermo Fisher Scientific) platform equipped with a 10x objective (Zeiss Plan Neofluar, NA 0.3 was used. Images were analyzed using the Compartmental Analysis Bio Application (Cellomics, Thermo Fisher Scientific). At least 500 valid objects were analyzed per well. Cell cycle analysis and analysis of cell loss were accomplished by using the Cell Cycle Bio Application (Cellomics, Thermo Fisher Scientific) using a minimum of 2,000 valid objects.

### Histone H2AX phosphorylation

HeLa cells were plated (2,000 cells/well) into each well of 384-well plates and incubated overnight at 37 °C under standard conditions. Cells were treated with test compounds for 6 hours under culturing conditions. Next, cells were fixed with 3.7% formaldehyde for 15 minutes and washed twice with DPBS. Fixed cells were permeabilized with 0.1% Triton X-100 in phosphate buffered saline (PBS) for 15 minutes and washed twice with DPBS. After blocking with 2% Fetal bovine serum (FBS, in DPBS) for 15 minutes cells were incubated with anti-Histone H2AX polyclonal antibody (PA184856-Thermo Scientific) for 1 hour. Samples were washed three times with wash buffer II (prepared in water) and twice with DPBS after which cells were incubated with Goat anti-mouse488 (GAM-DyLight 488) secondary antibody for 1 hour. Cells were washed three times with wash buffer II and nuclei were stained with Hoechst33342 (OG1726671-Thermo Scientific). Next, cells were washed twice with DPBS and a final volume of 25 μl DPBS was added. For the analysis of stained cells the CellomicsArrayScan VTI (Thermo Fisher Scientific) platform equipped with a 10x objective (Zeiss Plan Neofluar, NA 0.3 was used. Images were analyzed using the Compartmental Analysis Bio Application (Cellomics, Thermo Fisher Scientific). At least 500 valid objects were analyzed per well.

### HIV-1 RT activity

For the analysis of HIV-1 reverse transcriptase activities, the EnzChek^®^ Reverse Transcriptase Assay Kit (Molecular Probes, Life Technologies) was used according to the manufacturer’s instructions. PicoGreen fluorescence was analyzed in a SpectraMax reader (Molecular Devices, Sunnyvale, CA, USA) using the 480/520 nm filter set.

### Data analysis

Raw data from automated image analysis for each cytological feature were related to corresponding values from control wells where the control was set to 1. All cytological features of a given compound were combined to result in a cytological profile. All cytological profiles were subjected to hierarchical clustering using average linkage clustering with optimized gene leaf order and Pearson or Spearman rank correlation using Multi Experiment Viewer (MeV v4.9)^28^. Dose-response curves were generated in Prism (GraphPad). Chemical similarity plots and Tanimoto similarity (atom pair) scores were generated by ChemMine Tools (chemmine.ucr.edu). Briefly, multidimensional scaling of chemical similarity started with a matrix of all-against-all compound distances (Tanimoto coefficient) and then assigned coordinates for each compound in a low-dimensional space to represent the distances graphically in a scatter plot.

## Additional Information

**How to cite this article:** Kremb, S. and Voolstra, C. R. High-resolution phenotypic profiling of natural products-induced effects on the single-cell level. *Sci. Rep.* **7**, 44472; doi: 10.1038/srep44472 (2017).

**Publisher's note:** Springer Nature remains neutral with regard to jurisdictional claims in published maps and institutional affiliations.

## Supplementary Material

Supplementary Information
