# Supplementary material for: High-resolution phenotypic profiling of natural products-induced effects on the single-cell level
Source: Sci Rep. 2017 Mar 15;7:44472. doi: 10.1038/srep44472 (PMC5353608; doi:10.1038/srep44472)
Supplement: Supplementary Information [file srep44472-s1.pdf]

Supplementary Information

High-resolution phenotypic profiling of natural products-induced effects on the single-cell level

Stephan Kremb[1,*] and Christian R. Voolstra[1,*]

[1]Red Sea Research Center, Division of Biological and Environmental Science and Engineering, King Abdullah University of Science and Technology (KAUST), Thuwal 23955-6900, Saudi Arabia

*Corresponding Authors

Contact Information:

Stephan Kremb

sk6225@nyu.edu

Christian R Voolstra

christian.voolstra@kaust.edu.sa

Supplementary Results

Supplementary Table 1. List of 20 core features selected from 134 cellular features retrieved from our imaging-based High-Content Screening technology platform.

Short name	Core feature	Reagent
NFkB act	NFkB activation	antibody
p53 act	p53 activation	antibody
Casp9 act	Caspase 9 activation	antibody
Cell Area	Whole cell area	WGA488
Cell Shape	Wholes cell shape	WGA488
Nuc Int	Nuclear Total Intensity	Hoechst33342
Nuc Area	Nuclear Total Area	Hoechst33342
Nuc Shape	Nuclear Shape	Hoechst33342
Actin Int	Actin Total Intensity	Phalloidin 488
Actin Count	Actin Filament Count	Phalloidin 488
Tub Int	Tubulin Total Intensity	Antibody
Tub Count	Tubulin Filament Count	Antibody
Mito Int	Mitochondrial Total Intensity	Mitotracker
Mito Count	Mitochondrial Count	Mitotracker
ER Int	ER Total Intensity	ERtracker
ER Count	ER count	ERtracker
Lyso Int	Lysosome Total Intensity	Lysotracker
Lyso Count	Lysosome Count	Lysotracker
PM Int	Plasma Membrane Total Intensity	WGA488
PM Count	Plasma Membrane Total Intensity	WGA488

Supplementary Table 2. Collection of 124 poorly-characterized single compound natural products used for high-resolution cytological profiling to assess High-Content Screening technology platform.

IDNUMBER	WEIGHT	NAME
AN-970/40920732	206.2	5-hydroxy-7-methoxy-2-methyl-4H-chromen-4-one
AC-776/25122008	254.28	2'-Hydroxy-4'-methoxychalcone
AC-776/21258006	504.49	3',4',5-Trihydroxy-3,7-dimethoxyflavone, derivative of
AC-776/21258024	356.33	4',5,7-Trimethoxyisoflavone, derivative of
AC-776/21258029	344.32	3',4',5,7-Tetrahydroxy-3-methoxyflavone, derivative of
AC-776/41252599	476.52	Lonchocarpic acid, derivative of
AC-776/41252607	382.41	Robustic acid, derivative of
AO-774/41465644	402.62	3-(tetrahydro-2H-pyran-2-yloxy)pregnan-21-al
AO-774/41465653	257.24	4-O-Methylgalactose, derivative of
AO-774/41465654	613.76	N-[14-hydroxy-10,13-dimethyl-17-(5-oxo-2,5-dihydrofuran-3-yl)hexadecahydro-1H-cyclopenta[a]phenanthren-3-yl](3,4,5-trihydroxy-6-methoxytetrahydro-2H-pyran-2-yl)methanesulfonamide
AO-774/41465521	476.65	2-(acetyloxy)-1-{[3-(acetyloxy)-10,13-dimethylhexadecahydro-1H-cyclopenta[a]phenanthren-17-yl]methyl}ethyl acetate
AO-774/41465524	445.6	17-[2-(acetyloxy)-1-cyano-1-hydroxyethyl]-10,13-dimethylhexadecahydro-1H-cyclopenta[a]phenanthren-3-yl acetate
AO-774/41465536	430.63	methyl 4-[3-(acetyloxy)-10,12-dimethylgon-13(17)-en-17-yl]pentanoate
AO-774/41465560	518.64	3,7,12-Trihydroxycholan-24-oic acid, derivative of
AO-774/41465564	430.54	methyl 11,12-bis(acetyloxy)abieta-9(11),8(14),12-trien-20-oate
AO-774/41465568	390.6	[3-(tetrahydro-2H-pyran-2-yloxy)androstan-17-yl]methanol
AO-774/41465447	148.16	6-methyltetrahydro-2H-pyran-2,4,5-triol
AO-774/41465361	495.45	Prebediolone acetate, derivative of
AO-774/41465368	432.6	2-[3-(acetyloxy)-10,13-dimethyl-2,3,4,7,8,9,10,11,12,13,14,15,16,17-tetradecahydro-1H-cyclopenta[a]phenanthren-17-yl]-2-hydroxypropyl acetate
AO-774/41465380	387.54	3,21-Dihydroxypregnan-20-one, derivative of
AO-774/41465386	427.58	17-[2-(acetyloxy)-1-cyanoethylidene]-10,13-dimethylhexadecahydro-1H-cyclopenta[a]phenanthren-3-yl acetate
AO-774/41465396	390.52	methyl 3-(acetyloxy)-15-oxoandrostane-17-carboxylate
AO-774/41465404	374.52	3,16-Dihydroxypregn-5-en-20-one, Derivative of
AO-774/41465405	374.52	6b-(1-hydroxyethyl)-4a,6a-dimethyl-2,3,4,4a,4b,5,6,6a,6b,7a,8,8a,8b,9-tetradecahydro-1H-naphtho[2',1':4,5]indeno[1,2-b]oxiren-2-yl acetate
AO-774/41465416	424.6	17-{[(methylsulfonyl)oxy]methyl}androst-14-en-3-yl acetate
AO-774/41465417	398.54	3-[3-(acetyloxy)-10,13-dimethyl-2,3,4,5,6,7,8,9,10,11,12,13,16,17-tetradecahydro-1H-cyclopenta[a]phenanthren-17-yl]-2-oxopropanide-1-diazonium
AN-919/14028003	457.5	N-{2-(2-fluorophenyl)-1-[(6-oxo-7,11-diazatricyclo[7.3.1.0~2,7~]trideca-2,4-dien-11-yl)carbonyl]vinyl}benzamide
AN-919/14028004	459.57	N-{2-(3-methyl-2-thienyl)-1-[(6-oxo-7,11-diazatricyclo[7.3.1.0~2,7~]trideca-2,4-dien-11-yl)carbonyl]vinyl}benzamide
AN-919/14028005	483.52	N-{2-(1,3-benzodioxol-5-yl)-1-[(6-oxo-7,11-diazatricyclo[7.3.1.0~2,7~]trideca-2,4-dien-11-yl)carbonyl]vinyl}benzamide
AN-919/14028007	548.43	2-bromo-N-{2-(4-methoxyphenyl)-1-[(6-oxo-7,11-diazatricyclo[7.3.1.0~2,7~]trideca-2,4-dien-11-yl)carbonyl]vinyl}benzamide
AN-919/14028009	561.48	2-bromo-N-{2-[4-(dimethylamino)phenyl]-1-[(6-oxo-7,11-diazatricyclo[7.3.1.0~2,7~]trideca-2,4-dien-11-yl)carbonyl]vinyl}benzamide
AN-919/14028010	524.44	2-bromo-N-[1-[(6-oxo-7,11-diazatricyclo[7.3.1.0~2,7~]trideca-2,4-dien-11-yl)carbonyl]-2-(2-thienyl)vinyl]benzamide
AG-670/20238004	283.24	Nebularine, derivative of
AE-848/20954002	412.4	[6-(6-hydroxy-9H-purin-9-yl)-2,2-dimethyltetrahydrofuro[3,4-d][1,3]dioxol-4-yl]methyl benzoate
AI-372/20970054	228.25	Ononetin, derivative of
AA-504/21163099	382.45	1-[2-hydroxy-4,6-dimethoxy-3-(3-methyl-2-butenyl)phenyl]-3-(4-methoxyphenyl)-2-propen-1-one
AA-504/21163105	442.51	1-[2-hydroxy-4,6-dimethoxy-3-(3-methyl-2-butenyl)phenyl]-3-(2,4,6-trimethoxyphenyl)-2-propen-1-one
AA-504/21163113	344.36	2',4'-Dihydroxy-3,4-dimethoxychalcone, derivative of
AJ-091/33874001	208.21	2,3-Dihydro-5,7-dihydroxy-2,2,6-trimethyl-4H-1-benzopyran-4-one, derivative of
AJ-091/33874004	236.27	2,3-Dihydro-5,7-dihydroxy-2,2,6-trimethyl-4H-1-benzopyran-4-one, derivative of
AQ-152/42730379	306.44	3,5,25-Trihydroxyergostan-6-one
AE-641/00404017	530.74	19-(1,2-dihydroxy-1-methylethyl)-4,5,9,9,13-pentamethyl-21-oxo-20-oxahexacyclo[17.2.2.0~1,18~.0~4,17~.0~5,14~.0~8,13~]tricos-10-yl acetate
AE-641/00404027	526.8	3,20,28-Lupanetriol, derivative of

AG-205/06483018	332.31	2-amino-4-(4-hydroxyphenyl)-5-oxo-4H,5H-pyrano[3,2-c]chromene-3-carbonitrile
AG-205/06484030	442.26	ethyl 2-amino-4-(4-bromophenyl)-5-oxo-4H,5H-pyrano[3,2-c]chromene-3-carboxylate
AA-504/07224027	386.66	Cholest-5-en-3-ol
AN-956/21248007	258.23	Dendroflorin
AO-313/21254001	535.53	Podophyllotoxin, derivative of
AO-313/21254004	551.98	Podophyllotoxin, derivative of
AO-313/21254005	596.43	Podophyllotoxin, derivative of
AO-313/21254006	596.43	Podophyllotoxin, derivative of
AP-114/21255001	450.45	(4S)-4-Ethyl-4,9-dihydroxy-1H-pyrano[3',4':6,7]indolizino[1,2-b]chinolin-3,14(4H,12H)-dion
AK-693/40757284	556.48	Morelloflavone
AN-919/14028002	482.58	N-{2-[4-(dimethylamino)phenyl]-1-[(6-oxo-7,11-diazatricyclo[7.3.1.0~2,7~]trideca-2,4-dien-11-yl)carbonyl]vinyl}benzamide
AI-372/20970048	355.39	N-[2-(2,3-dihydro-1,4-benzodioxin-6-yl)-4H-chromen-4-ylidene]-N-phenylamine
AI-372/21056009	355.35	Nebularine, derivative of
AA-504/21163120	342.31	Demethoxykanugin, derivative of
AA-504/21163123	358.35	3-Hydroxy-3',4',5,5',7-pentamethoxyflavone, derivative of
AK-693/21212018	246.31	Isofischeric acid
AK-693/21212019	554.68	Acrovestone
AO-229/21213002	424.49	Dorsmanin E
AO-229/21213022	368.39	Cannflavin B
AO-229/21213024	392.49	Stipulin
AO-313/21215010	544.56	Podophyllotoxin, derivative of
AO-313/21215011	439.42	Podophyllotoxin, derivative of
AO-313/21215013	489.91	Podophyllotoxin, derivative of
AO-313/21215014	562.53	Podophyllotoxin, derivative of
AO-313/21215015	562.53	Podophyllotoxin, derivative of
AO-313/21215017	543.57	Podophyllotoxin, derivative of
AO-313/21215019	551.98	Podophyllotoxin, derivative of
AO-313/21215020	596.43	Podophyllotoxin, derivative of
AO-656/21226005	368.47	8,13-Epoxy-1,6,7,9-tetrahydroxy-14-labden-11-one
AJ-738/21233004	374.35	3',6-Dihydroxy-3,4',5,7-tetramethoxyflavone
AE-641/00404015	513.72	19-(N-hydroxyethanimidoyl)-4,5,9,9,13-pentamethyl-21-oxo-20-oxahexacyclo[17.2.2.0~1,18~.0~4,17~.0~5,14~.0~8,13~]tricos-10-yl acetate
AO-313/21254002	535.53	Podophyllotoxin, derivative of
AP-063/21256002	360.32	3',4',5,6,7-Pentahydroxy-3-methoxyflavone, derivative of
AN-308/21259003	256.26	2,4,4'-Trihydroxychalcone
AP-157/40757271	528.52	Shiraiachrome C
AO-774/41465496	254.24	2-oxo-3-(3,4,5-trimethoxyphenyl)propanoic acid
AO-774/41465379	345.51	3,21-Dihydroxypregnan-20-one, derivative of
AO-774/41465402	464.56	methyl 2,7-bis(acetyloxy)-5a-hydroxy-9a,11a-dimethylhexadecahydronaphtho[1',2':6,7]indeno[1,7a-b]oxirene-1-carboxylate
AJ-292/41630391	459.58	3'-[(acetyloxy)methyl]-3'-(aminocarbonyl)-spiro[androst-5-ene-17,2'-oxirane]-3-yl acetate
AN-919/14028006	445.54	N-[(E)-1-[(6-oxo-7,11-diazatricyclo[7.3.1.0~2,7~]trideca-2,4-dien-11-yl)carbonyl]-2-(2-thienyl)ethenyl]benzamide
AN-919/14028008	578.46	2-bromo-N-{2-(2,3-dimethoxyphenyl)-1-[(6-oxo-7,11-diazatricyclo[7.3.1.0~2,7~]trideca-2,4-dien-11-yl)carbonyl]vinyl}benzamide
AI-899/21033047	424.49	Sigmoidin A
AA-504/21163091	510.63	Kenusanone B, derivative of
AA-504/21163118	314.34	2',4'-Dihydroxy-3,4-dimethoxychalcone, derivative of
AA-504/21163124	312.32	3',4'-Dimethoxyflavanol, derivative of
AH-214/21165009	474.42	7-O-beta-D-Glucuronopyranoside, derivative of
AK-693/21212006	562.79	Carpaine, derivative of
AO-313/21215001	547.56	Podophyllotoxin, derivative of
AK-025/40890504	236.31	9-Deoxymuzigadial, derivative of
AK-693/21164013	203.24	Ent-Norsecurinine
AK-693/40760140	336.34	3,9-Dihydroxy-2-(3-methyl-2-buten-1-yl)-6H-[1]benzofuro[3,2-c]chromen-6-on
AO-313/21215016	531.56	Podophyllotoxin, derivative of
AJ-738/20210002	584.71	(1a,14a,16b)-20-Ethyl-1,14,16-trimethoxyaconitane-4,8,9-triol 4-[2-(Acetylamino)benzoate]
AK-693/21164005	462.45	Exoticin
AK-693/40881553	230.26	7-Prenyloxycoumarin
AK-693/40962738	284.31	5-Hydroxy-7-methoxy-8-methylflavanone
AK-693/40962756	296.36	Attenuol
AK-693/40962758	293.36	Koenimbine
AK-693/41507039	258.27	Phebalosin
AK-693/41507040	206.24	p-Coumaric acid methyl ether ethyl ester
AK-693/41507054	368.38	Kaerophyllin
AK-693/43417381	197.24	1-Hydroxy-3-methyl-9H-carbazole

AK-693/43417382	222.24	Verticilone
AK-693/43417391	222.2	7-Hydroxy-5,8-dimethoxycoumarin
AK-693/43417398	432.42	3,3',4',5,5',7,8-Heptamethoxyflavone
AM-730/20761034	317.34	1-[(2E)-3-(3,4,5-Trimethoxyphenyl)-2-propenoyl]-5,6-dihydro-2(1H)-pyridinon
AP-163/40806740	280.41	Ibogamine
AK-693/40962743	326.3	Demethoxykanugin
AG-670/20238030	357.37	2-[6-(benzylamino)-9H-purin-9-yl]-5-(hydroxymethyl)tetrahydro-3,4-furandiol
AH-262/20354047	491.58	4-amino-5-[2-(11,17-dihydroxy-10,13-dimethyl-3-oxo-2,3,6,7,8,9,10,11,12,13,14,15,16,17-tetradecahydro-1H-cyclopenta[a]phenanthren-17-yl)-2-oxoethoxy]-5-oxopentanoic acid
AK-693/40962741	188.18	Psoralen, derivative of
AK-693/40962736	265.35	Dihydroxygirinimbine, derivative of
AK-693/40962763	527.81	3-[(aminocarbothioyl)hydrazono]lanosta-7,24-dien-26-oic acid
AK-820/13220073	381.51	Embelin, derivative of
AK-693/40962724	392.45	Curcumin, derivative of
AO-801/40870156	404.63	1-(1,5-dimethylhexyl)-7a-methyl-5-(1-methyl-2-oxocyclohexyl)octahydro-1H-indene-4-carboxylic acid
AA-504/21163076	324.42	1,7-bis(4-methoxyphenyl)-4-hepten-3-one
AO-313/21254008	643.42	Podophyllotoxin, derivative of
AK-693/40962739	288.3	Ceylantin
AI-372/20970063	420.41	Onospin
AP-163/40806890	200.1	Poppy acid

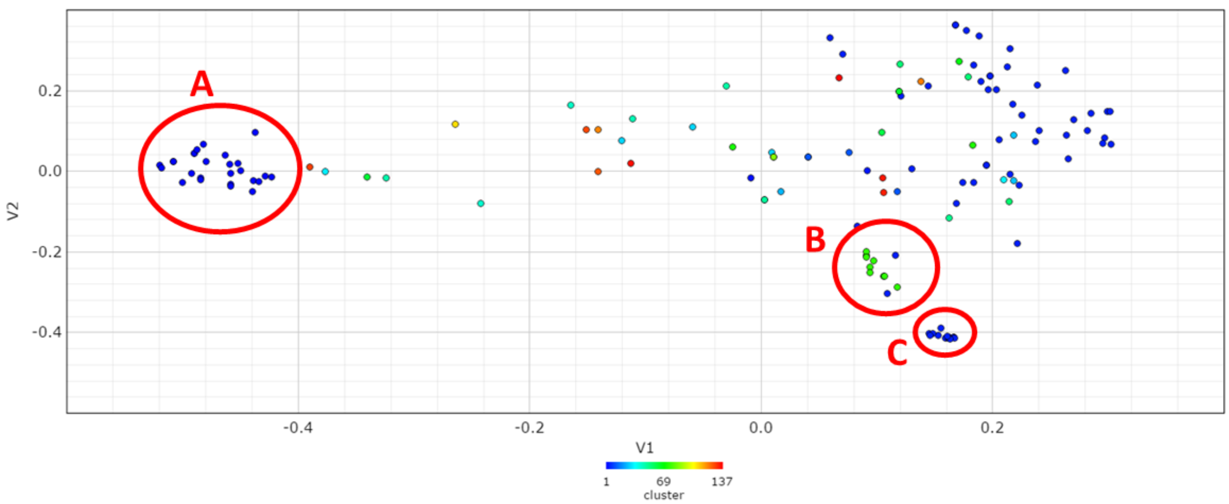

Supplementary Figure 1. Multi-dimensional scaling (MDS) plot of structural similarities of a collection of 124 natural products assessed in this study. Red circles indicate groups of related chemical structures (A = steroid scaffolds; B = derivatives of podophyllotoxin; C = derivatives of a diazatricyclo scaffold). Chemical similarities were generated and plotted by ChemMine Tools (chemmine.ucr.edu).

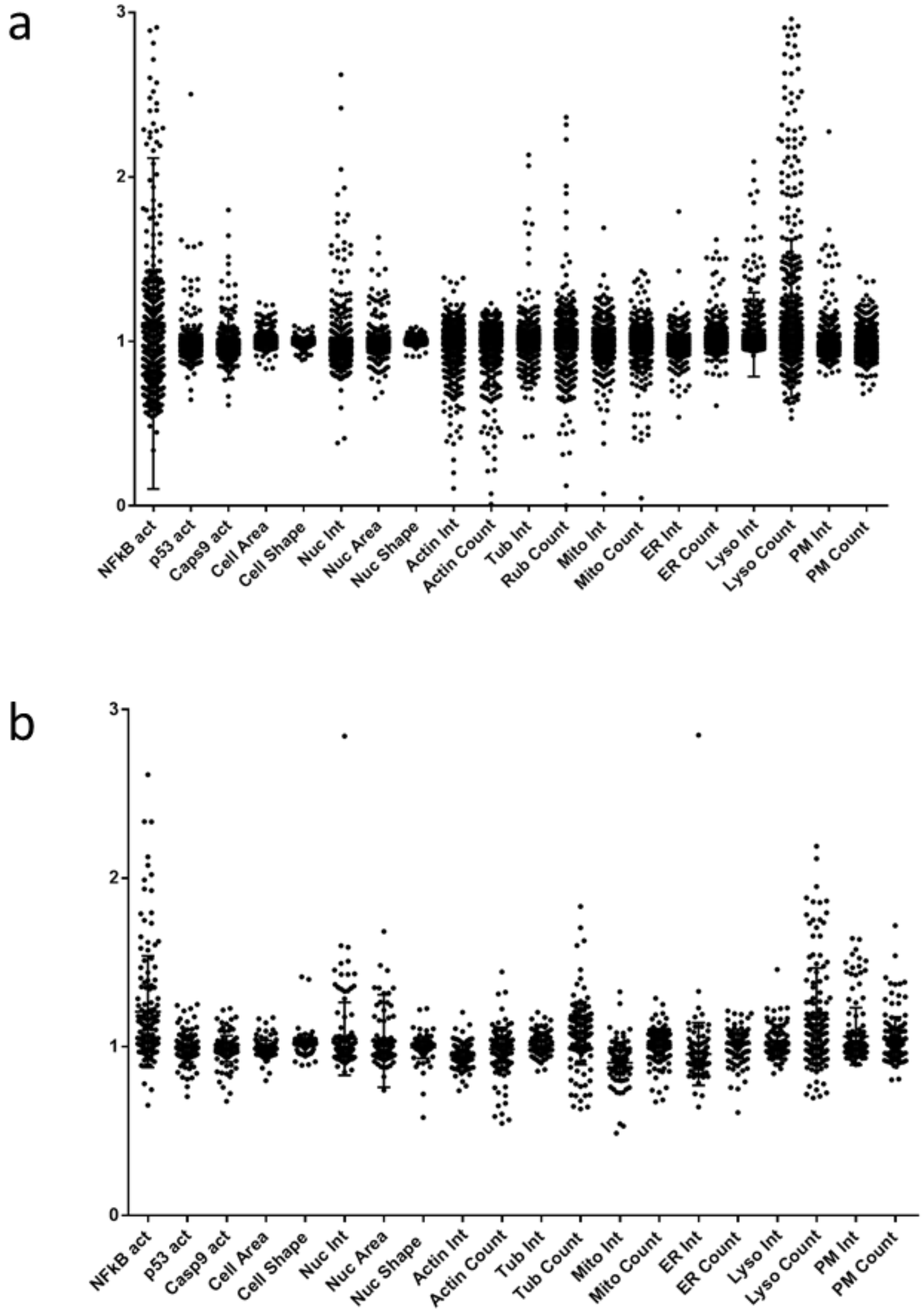

Supplementary Figure 2. Dot plots depicting the relative distribution of compound-induced perturbations on the set of 20 core features (see Supplementary Table S1). Values of 1 indicate no effect. a) Collection of 720 bioactive reference compounds with known mechanism of actions. b) Collection of 124 natural products.

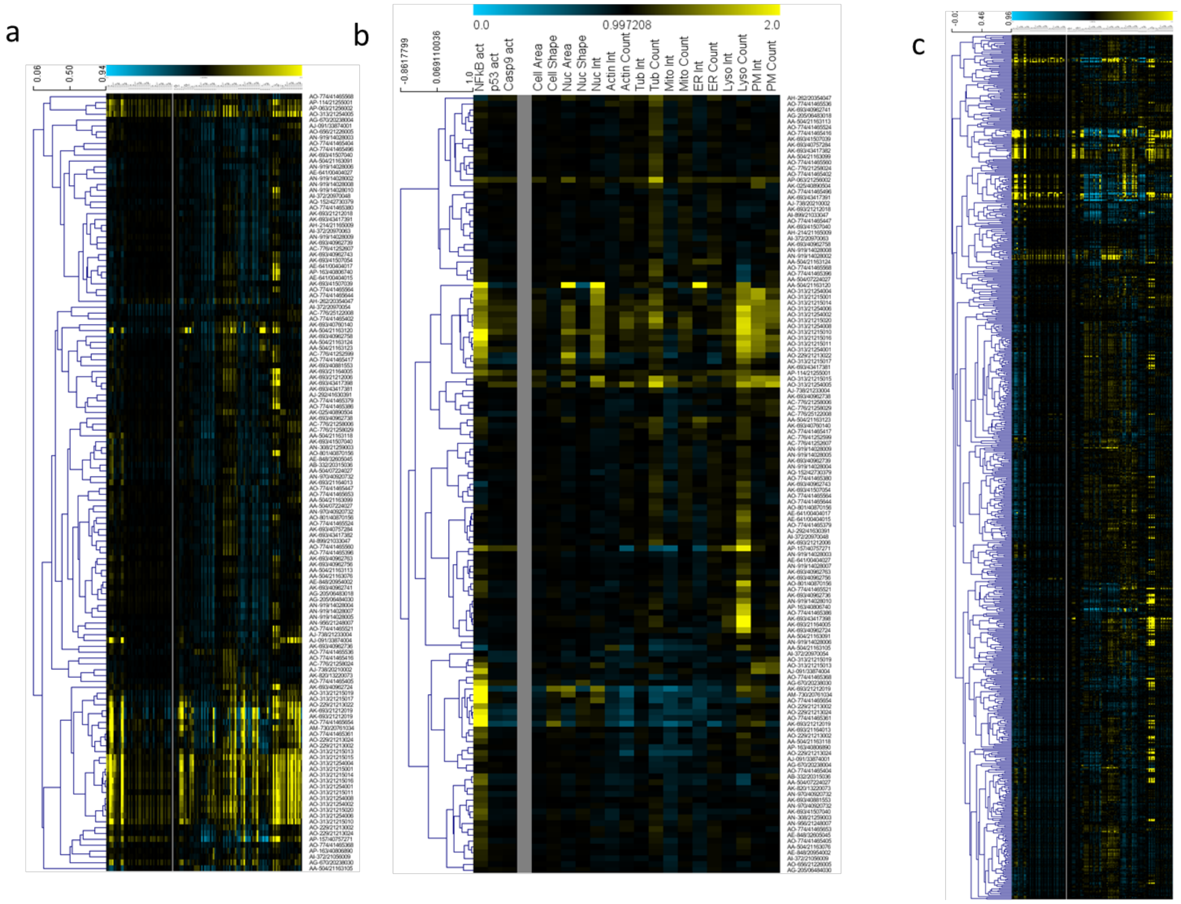

Supplementary Figure 3. Cluster analysis of cytological profiles composed of 134 cellular descriptors obtained from (a) a collection of 124 poorly characterized natural products and (c) a set of 720 reference compounds with assigned mechanisms of actions at a concentration of 10 µM. For better visualization, a set of (b) 20 core features (Supplementary Table 1) was used for cluster analysis of the natural products collection, and a vertical grey bar was used to separate between regulatory (i.e. NFkB, p53 and caspase 9 activation) and other cellular markers. Colors indicate positive (yellow) or negative (blue) deviation from the mean of untreated control cells for each cellular feature (value = 1). The dendrogram depicts distances between individual cytological profiles based on Spearman rank correlation.

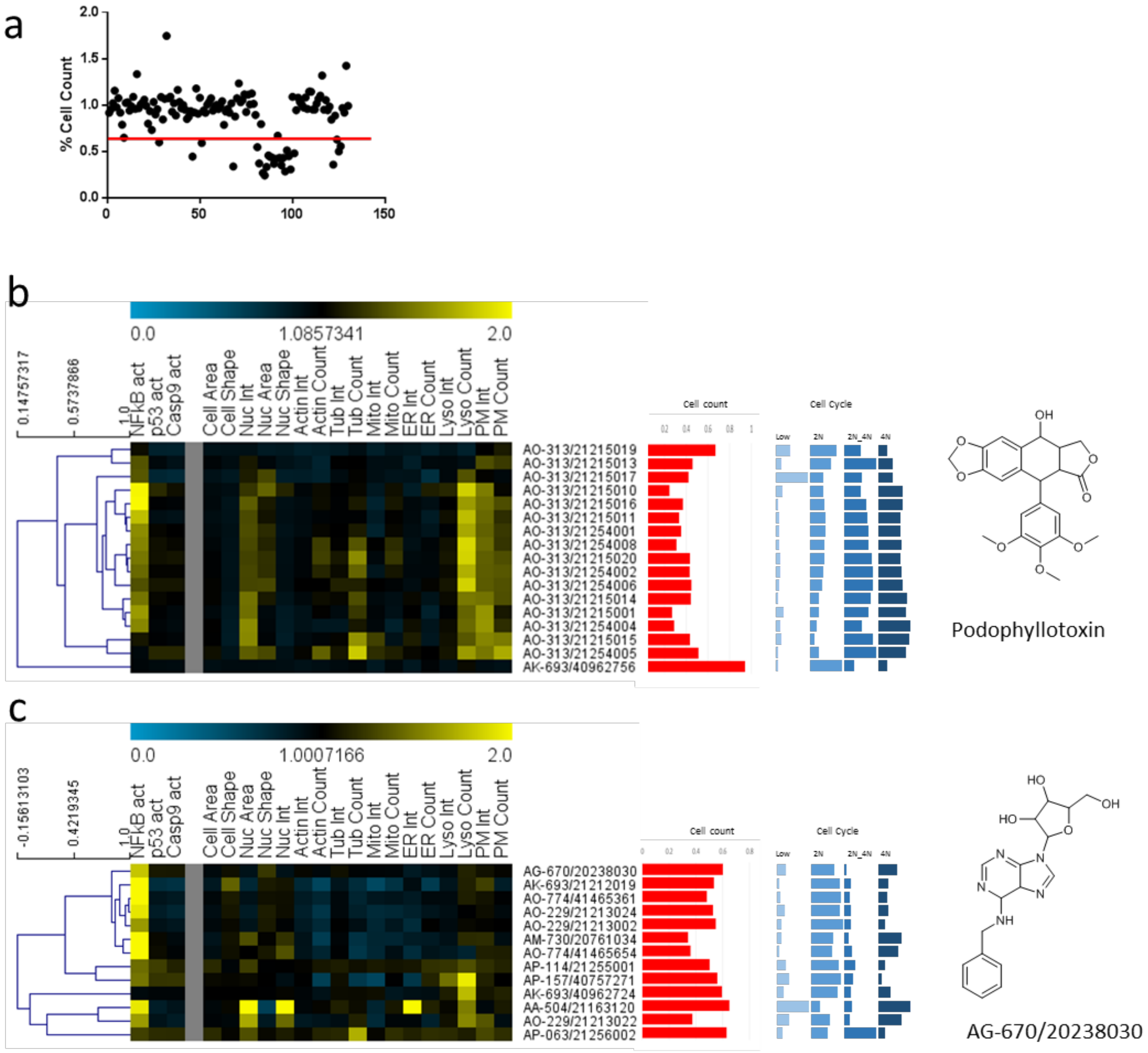

Supplementary Figure 4. Analysis of candidate toxic compounds from a collection of 124 poorly characterized natural products. a) 30 natural products were found to reduce cell counts by more than 40 percent compared to control-treated wells. b) A group of 16 derivatives of podophyllotoxin were found to cause strong reductions of cell counts (red bars) and also lead to consistent mitotic arrest of the cell cycle (blue bars). Cytological profiles show consistent perturbations of a set of core features as well as individual differences. c) A group of structurally unrelated natural products inducing

reduced cell counts show various distinct cytological profiles, including a group of highly similar profiles with strong activation of NFkB and negative perturbations of actin, tubulin, and mitochondrial markers. Red bars depict cell counts and blue bars show relative numbers of cells in each phase of the cell cycle. AG-670/20238030 = 6-phenyladenosine. Cytological profiles consisting of a reduced set of 20 core markers were used for easier visualization of affected processes and a vertical grey bar was used to separate between regulatory (i.e. NFkB, p53 and caspase 9 activation) and other cellular markers. Colors indicate positive (yellow) or negative (blue) deviation from the mean of untreated control cells for each cellular feature (value = 1). The dendrogram depicts distances between individual cytological profiles based on Pearson correlation.

Supplementary Text 1. Prediction and validation of biological targets/mechanisms of actions (MOAs) of antiviral compounds

The plant-derived kenunsanone B was found to closely match with two protease inhibitors, E-64 and nelfinavir (NLV) (Supplementary Fig. 5a). While NLV is a potent inhibitor of the HIV-1 protease (an aspartate protease), E-64 is a selective inhibitor of cysteine proteases. Using a biochemical HIV-1 protease assay we found strong inhibitory activities of the candidate molecule and NLV, whereas E-64 showed no inhibitory effect at the same concentration range (Supplementary Fig. 5b). Interestingly, NLV and Ritonavir (RTV) exhibit different CPs compared to a set of related HIV-1 protease inhibitors. Using a higher concentration (20 μM), these differences are even more pronounced and most severely affect nuclear parameters as well as activation of NFkB and ER-related markers (Supplementary Fig. 5c). This is in accordance with previous accounts of NLV-related off-target effects as an anti-cancer drug with multiple effects, including ER stress and NFkB activation[1]. Importantly, NLV was recently suggested as a potential therapeutic for the treatment of mTORC-1 driven tumors[2]. NLV contains a characteristic S-phenyl group at P1 while the isopropyl thiazolyl P3 group in RTV is longer than in the other FDA-approved protease inhibitors. The increased ER activity is reflected in the CPs of NLV and RTV. In addition, the slightly increased membrane signal might be related to the influence of these compounds on lipoprotein metabolism[1]. Moreover, the cytological profiles of NLV and RTV matched closely to two other reference compounds with known anti-proliferative effects, PMA and mevastatin[3,4]. Our findings on antiviral compounds suggest that cytological profiling can provide a promising open-target strategy for prioritizing molecules for further studies on pathogens without the need of utilizing viruses or other pathogenic organisms in the primary screening campaign.

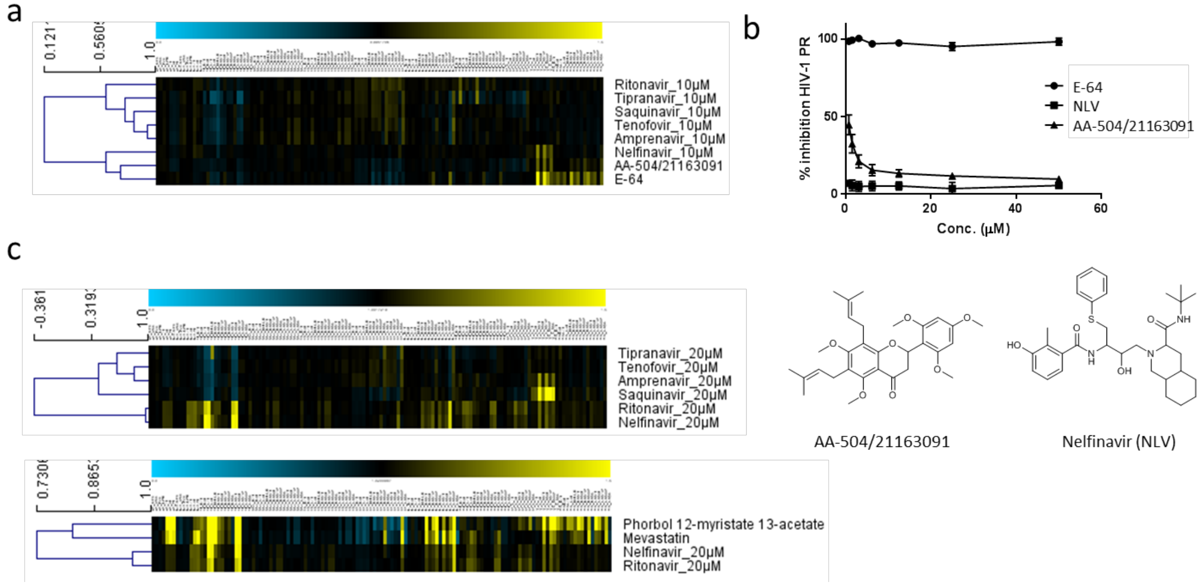

Supplementary Figure 5. Prediction and validation of biological targets/ mechanisms of actions (MOAs) of antiviral compounds. a) Hierarchical clustering of the full cytological profiles of a group of six protease inhibitors and one natural product candidate compound reveals grouping of the candidate compound with a serine protease inhibitor (E-64) and nelfinavir (NLV), an HIV-1 protease inhibitor. b) Both reference protease inhibitors and the candidate compounds were tested for activity on the HIV-1 protease in a biochemical assay. Whereas NLV and the candidate compound show strong inhibition at the tested concentration range (i.e., 1 – 50 µM), E-64 was found to be inactive. c) Hierarchical clustering of the cytological profiles captured at a higher compound concentration (20 µM) of a group of six HIV-1 protease inhibitors reveals two subgroups distinguishing NLV and ritonavir (RTV) from the remainder of the compounds. The cytological profiles of NLV and RTV were found to match closely with two reference compounds with assigned anti-proliferative activity (PMA and mevastatin). Colors indicate positive (yellow) or negative (blue) deviation from the mean of untreated control cells (value = 1). Heatmap clustering is based on Pearson correlation.

Supplementary Text 2. Prediction of biological targets/mechanisms of actions (MOAs) of neurotransmitter-related compounds.

A set of structurally diverse NP candidate molecules were found clustering within a large group of neurotransmitter (NT)-related reference compounds, including NT receptor antagonists, NT re-/uptake inhibitors, or ion channel interfering compounds (Supplementary Figs. 6a, b). Notably, most of the candidate molecules show clear structural similarities to several of the reference compounds (Supplementary Fig. 6c). The cytological profiles of all of these compounds are characterized by a strong increase of lysosomes counts. A number of drugs found in this cluster are well known to accumulate in lysosomes, e.g. fluoxetine, chlorpromazine, chloroquine, clomipramine or desipramine[5]. Lysosomotropism of drug molecules may lead to a marked increase of lysosomes and has several important implications including multidrug resistance (MDR), autophagy, or shingomyelinase activity[6-8].

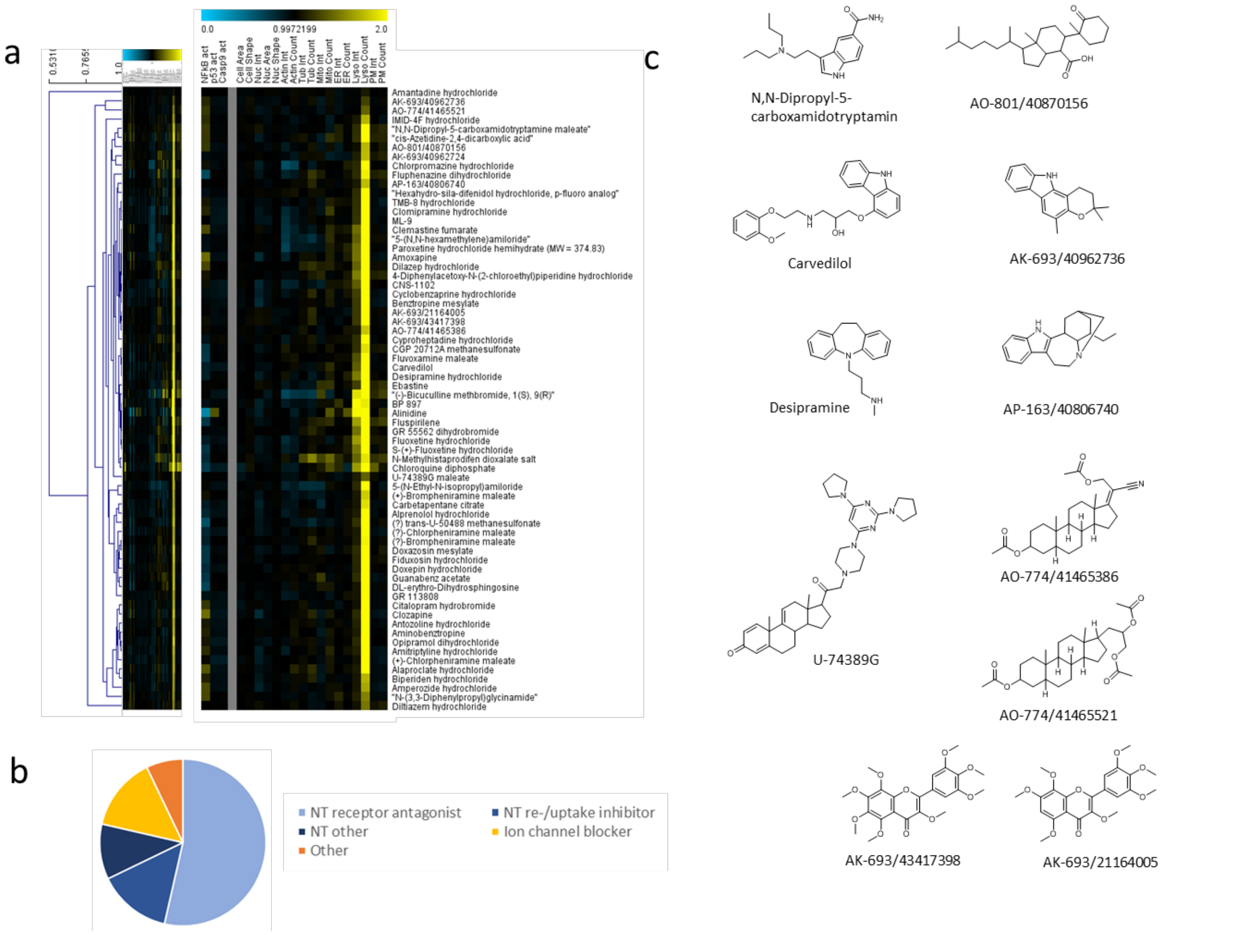

Supplementary Figure 6. Prediction of biological targets/mechanisms of actions (MOAs) of neurotransmitter-related compounds. a) Hierarchical clustering of the cytological profiles of all reference and candidate compounds revealed a large group of closely matching compounds characterized by a strong increase of lysosomal counts. Full cytological profiles were used for clustering, but only the reduced set of 20 core features are displayed for easier visualization of affected processes. A vertical grey bar was used to separate between regulatory (i.e. NFkB, p53 and caspase 9 activation) and other cellular markers. Colors indicate positive (yellow) or negative (blue) deviation from the mean of untreated control cells (value = 1). Heatmap clustering is based on Pearson correlation. b) More than 80% of the reference compounds found in this group are related to neurotransmitter (NT) activity. c) Candidate molecules from the natural production collection found in this group exhibit clear structural similarities to several of the reference compounds.

Supplementary Text 3. High-resolution cell-based structure-activity profiling of compounds containing steroid scaffolds

To further explore structure-activity relationships a set of 18 natural products with a steroid scaffold as well as three structurally related reference compounds were clustered (Supplementary Fig. 7a). Two major clusters were retrieved with several molecules showing strong perturbations on a characteristic set of markers. These molecules included two reference compounds, dihydroouabin and 2-methoxyestradiol as well as three molecules from our natural product collection. The reference compounds do not share much structural similarity but show high phenotypic similarity including strong activation of NFkB, increased nuclear intensity as well as strongly negative perturbation of actin features. However, they differ with regard to mitochondrial membrane potential and lysosomal counts as well as plasma membrane related markers. In contrast, dihydroouabin, a cardiac glycoside with an inhibitory activity on the sodium-potassium pump[9], shares structural and phenotypic similarity with one of the candidate molecules of the NP collection (Supplementary Fig. 7b). Another set of steroid molecules were found to exhibit low overall perturbations but consistently show a fairly strong induction of tubulin markers. One of the reference compounds, beclomethansone which is an anti-inflammatory glucocorticoid[10], shares this effect on tubulin but also exhibits a moderate activation of NFkB as well as a negative perturbation of lysosomal counts. Similar effects were also found for two structurally unrelated candidate molecules from our NP collection.

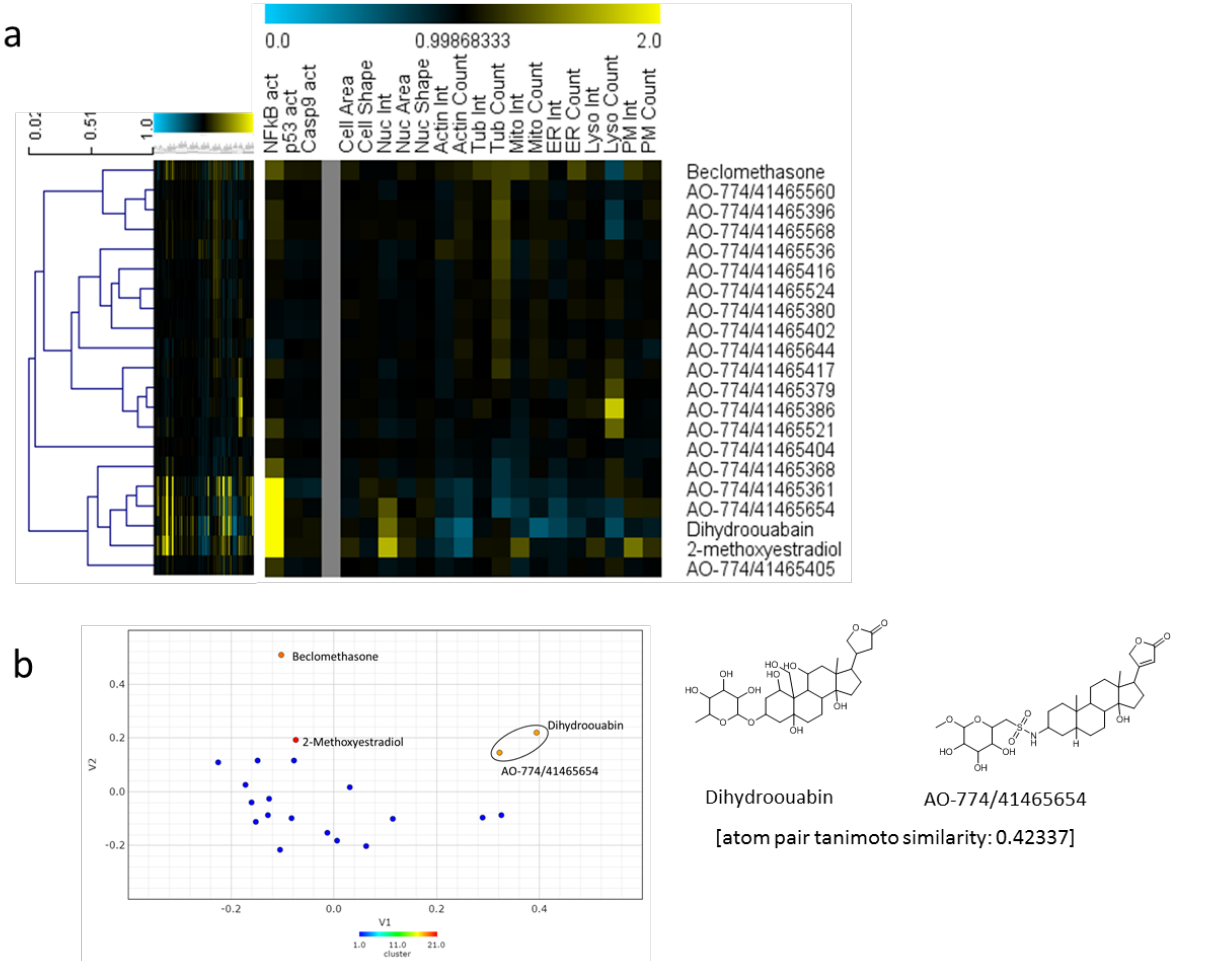

Supplementary Figure 7. High-resolution cell-based structure-activity profiling of compounds containing steroid scaffolds. a) Hierarchical clustering of the cytological profiles of a group of 18 natural products and 3 reference compounds sharing steroid scaffolds. Full cytological profiles were used for clustering, but only the reduced set of 20 core features is displayed for easier visualization of affected processes. A vertical grey bar was used to separate between regulatory (i.e. NFkB, p53 and caspase 9 activation) and other cellular markers. Colors indicate positive (yellow) or negative (blue) deviation from the mean of untreated control cells (value = 1). Heatmap clustering is based on Pearson correlation. b) Multi-dimensional scaling (MDS) plot of structural similarities of all tested compounds containing steroid scaffolds. The circle indicates a reference/NP compound pair sharing similar chemical structural elements.

Support Trees

For hierarchical trees depicted in Figures 2, 3, S4, S5, S6 and S7, we generated support trees that show statistical support for the nodes of the trees, based on resampling the data by bootstrapping (resampling with replacement). The numbers shown denote the percentage of times a given node was supported over the resampling trials.

Support tree for Figure 2

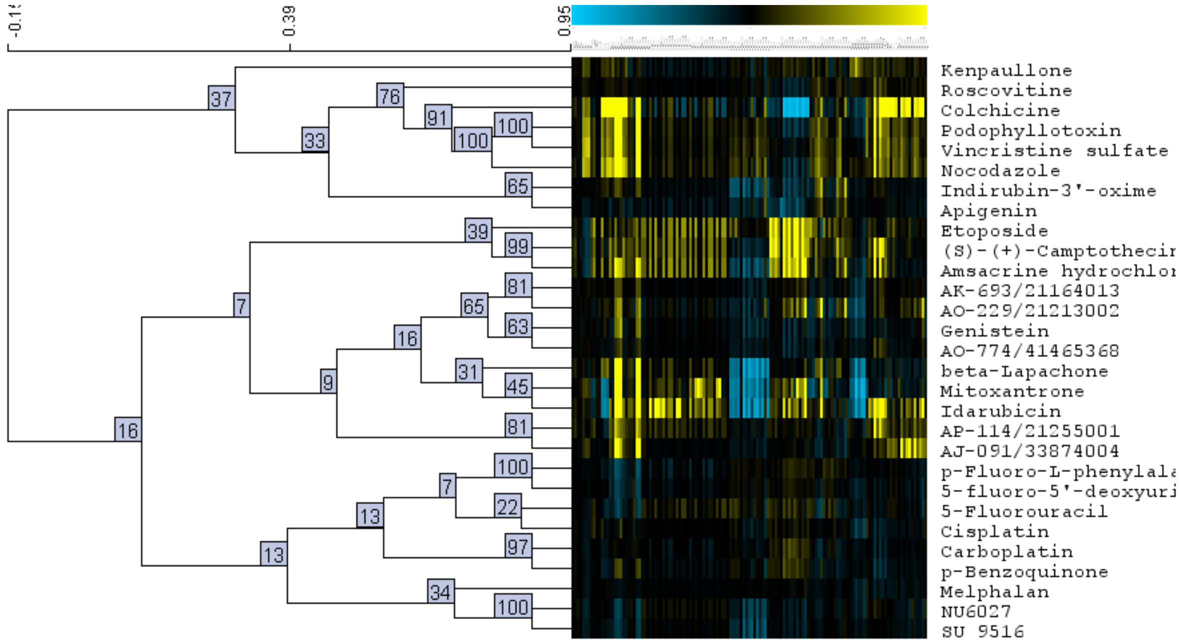

Support tree for Figure 3

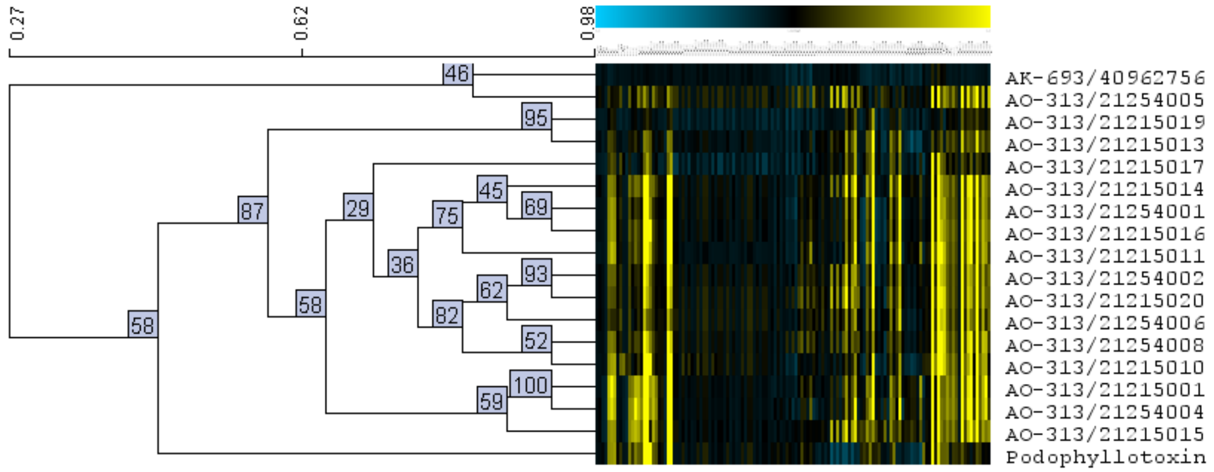

Support tree for Figure S4B

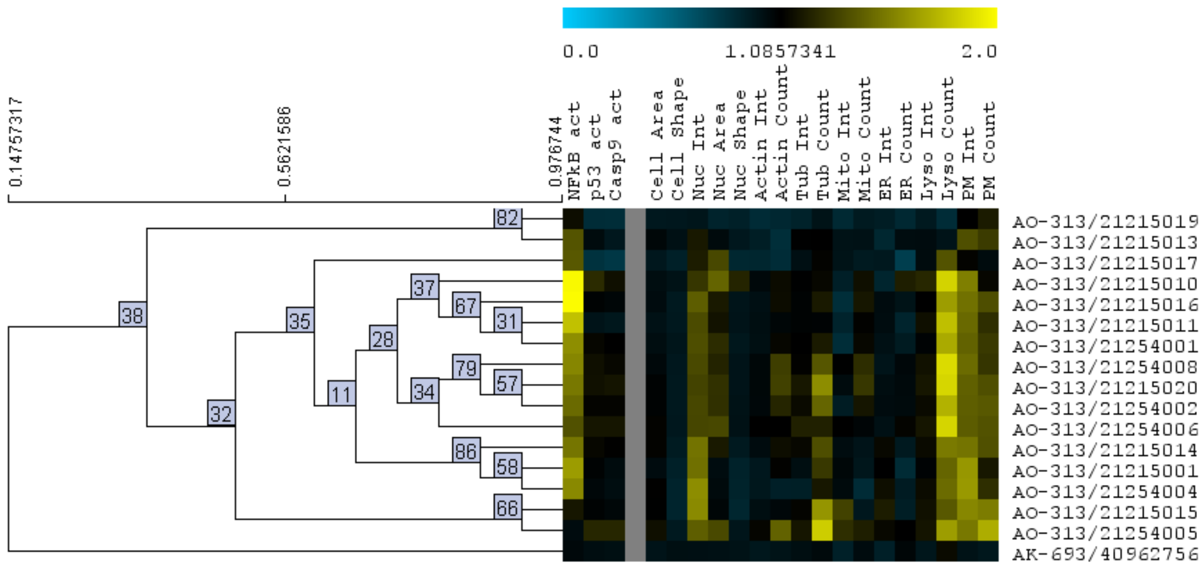

Support tree for Figure S4C

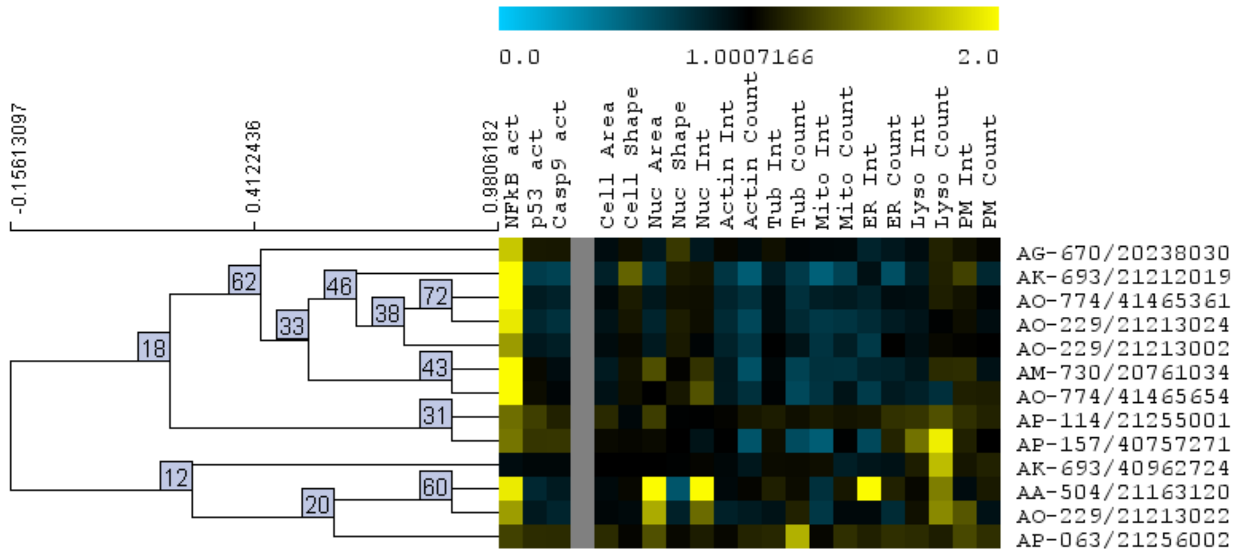

Support tree for Figure S5B

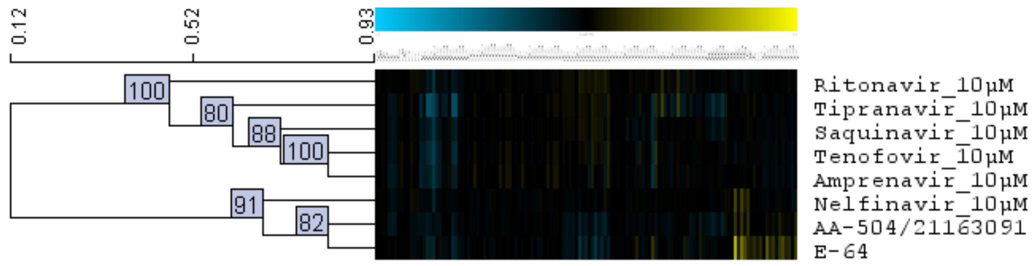

Support trees for Figure S5C

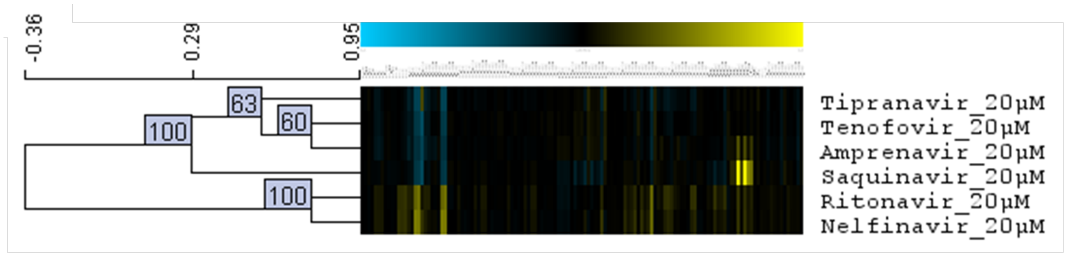

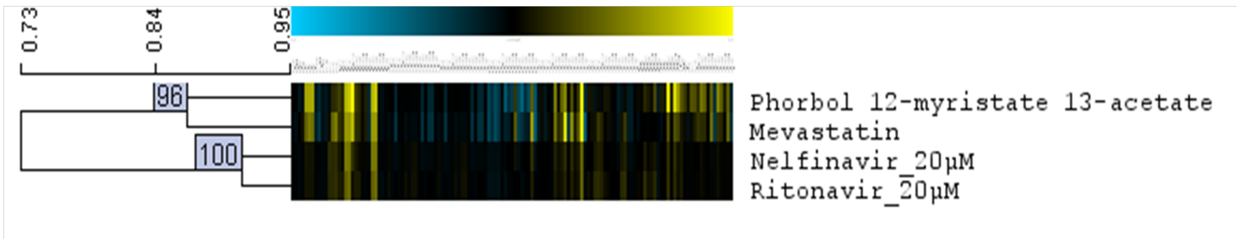

Support tree for Figure S6

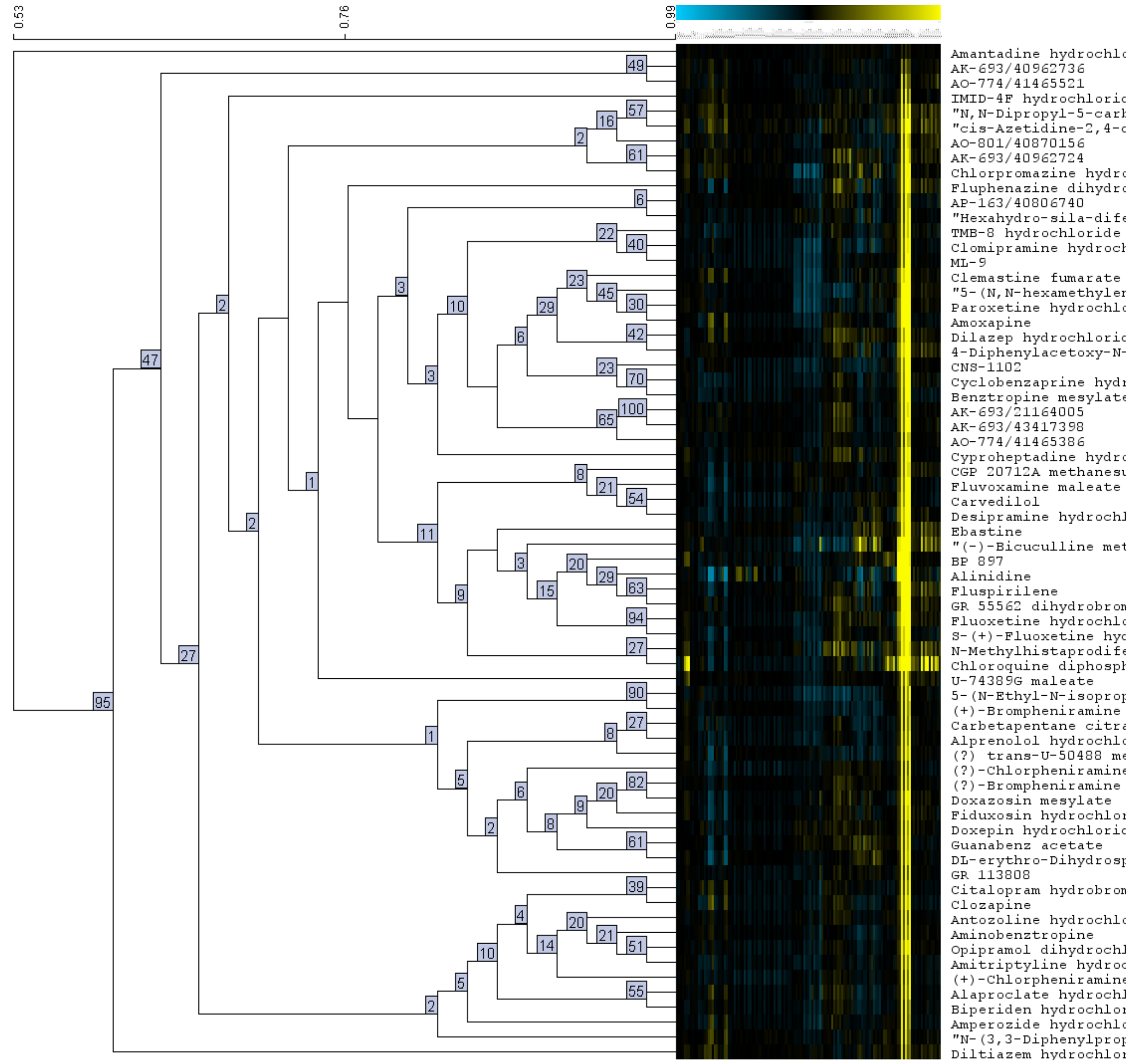

Support tree for Figure S7

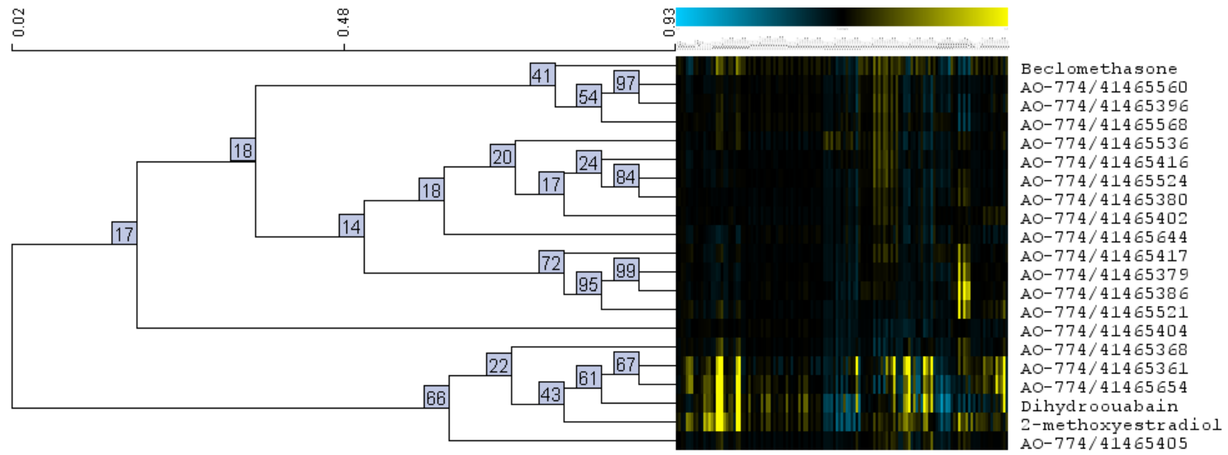

Supplementary References

1. Koltai, T. Nelfinavir and other protease inhibitors in cancer: mechanisms involved in anticancer activity. *F1000Research* **4**, 9 (2015).
2. Johnson, C.E. et al. Endoplasmic reticulum stress and cell death in mTORC1-overactive cells is induced by nelfinavir and enhanced by chloroquine. *Molecular oncology* **9**, 675-688 (2015).
3. Chaman, N., Iqbal, M.A., Siddiqui, F.A., Gopinath, P. & Bamezai, R.N. ERK2-Pyruvate Kinase Axis Permits Phorbol 12-Myristate 13-Acetate-induced Megakaryocyte Differentiation in K562 Cells. *The Journal of biological chemistry* **290**, 23803-23815 (2015).
4. Glynn, S.A., O'Sullivan, D., Eustace, A.J., Clynes, M. & O'Donovan, N. The 3-hydroxy-3-methylglutaryl-coenzyme A reductase inhibitors, simvastatin, lovastatin and mevastatin inhibit proliferation and invasion of melanoma cells. *BMC cancer* **8**, 9 (2008).
5. Trapp, S., Rosania, G.R., Horobin, R.W. & Kornhuber, J. Quantitative modeling of selective lysosomal targeting for drug design. *European biophysics journal : EBJ* **37**, 1317-1328 (2008).
6. Zhitomirsky, B. & Assaraf, Y.G. Lysosomal sequestration of hydrophobic weak base chemotherapeutics triggers lysosomal biogenesis and lysosome-dependent cancer multidrug resistance. *Oncotarget* **6**, 1143-1156 (2015).
7. Zhitomirsky, B. & Assaraf, Y.G. Lysosomes as mediators of drug resistance in cancer. *Drug resistance updates : reviews and commentaries in antimicrobial and anticancer chemotherapy* **24**, 23-33 (2016).
8. Kornhuber, J. et al. Identification of new functional inhibitors of acid sphingomyelinase using a structure-property-activity relation model. *Journal of medicinal chemistry* **51**, 219-237 (2008).
9. Mogul, D.J., Rasmussen, H.H., Singer, D.H. & Ten Eick, R.E. Inhibition of Na-K pump current in guinea pig ventricular myocytes by dihydroouabain occurs at high- and low-affinity sites. *Circulation research* **64**, 1063-1069 (1989).
10. Schmidt, J., Fleissner, S., Heimann-Weitschat, I., Lindstaedt, R. & Szelenyi, I. The effect of different corticosteroids and cyclosporin A on interleukin-4 and interleukin-5 release from murine TH2-type T cells. *European journal of pharmacology* **260**, 247-250 (1994).
